# Particulate matters increase epithelial-mesenchymal transition and lung fibrosis through the ETS-1/NF-κB-dependent pathway in lung epithelial cells

**DOI:** 10.1186/s12989-020-00373-z

**Published:** 2020-08-14

**Authors:** Yu-Chen Chen, Tzu-Yi Chuang, Chen-Wei Liu, Chi-Wei Liu, Tzu-Lin Lee, Tsai-Chun Lai, Yuh-Lien Chen

**Affiliations:** 1 Department of Anatomy and Cell Biology, College of Medicine, National Taiwan University, No. 1, Sec 1, Jen-Ai Road, Taipei, Taiwan, Republic of China; 2 Division of Pulmonary Medicine, Department of Internal Medicine, Min-Sheng General Hospital, No. 168 Ching-Kuo Road, Taoyuan, Taiwan, Republic of China; 3 Department of Internal Medicine, College of Medicine and National Taiwan University Hospital, No.7, Chung-Shan South Road, Taipei, Taiwan, Republic of China; 4 Department of Basic Medical Science, University of Arizona College of Medicine, Phoenix, AZ USA; 5 Department of Internal Medicine, Taoyuan General Hospital, Ministry of Health and Welfare, Taoyuan, Taiwan, Republic of China

**Keywords:** Particulate matters (PMs), EMT, Fibronectin, ETS-1, Pulmonary fibrosis

## Abstract

**Background:**

Particulate matters (PMs) in ambient air pollution are closely related to the incidence of respiratory diseases and decreased lung function. Our previous report demonstrated that PMs-induced oxidative stress increased the expression of proinflammatory intracellular adhesion molecule-1 (ICAM-1) through the IL-6/AKT/STAT3/NF-κB pathway in A549 cells. However, the role of O-PMs in epithelial-mesenchymal transition (EMT) development and pulmonary fibrosis and the related mechanisms have not been determined. The aim of this study was to investigate the effects of O-PMs on the pathogenesis of EMT and pulmonary fibrosis as well as the expression of ETS-1 and NF-κB p65, in vitro and in vivo.

**Results:**

O-PMs treatment induced EMT development, fibronectin expression, and cell migration. O-PMs affected the expression of the EMT-related transcription factors NF-κB p65 and ETS-1. Interference with NF-κB p65 significantly decreased O-PMs-induced fibronectin expression. In addition, O-PMs affected the expression of fibronectin, E-cadherin, and vimentin through modulating ETS-1 expression. ATN-161, an antagonist of integrin α5β1, decreased the expression of fibronectin and ETS-1 and EMT development. EMT development and the expression of fibronectin and ETS-1 were increased in the lung tissue of mice after exposure to PMs for 7 and 14 days. There was a significant correlation between fibronectin and ETS-1 expression in human pulmonary fibrosis tissue.

**Conclusion:**

O-PMs can induce EMT and fibronectin expression through the activation of transcription factors ETS-1 and NF-κB in A549 cells. PMs can induce EMT development and the expression of fibronectin and ETS-1 in mouse lung tissues. These findings suggest that the ETS-1 pathway could be a novel and alternative mechanism for EMT development and pulmonary fibrosis.

## Introduction

Fine particulate matter (PM) from the environment is easily inhaled into the respiratory tract, accumulates and penetrates into alveolar cells, and may result in structural damage and functional impairment of the respiratory system [1]. PM can potentially exacerbate pre-existing pulmonary disorders such as asthma, chronic obstructive pulmonary disease (COPD), pulmonary fibrosis, and even cancer [2]. Several mechanisms have been suggested to be involved in the adverse lung effects of PM, including cytotoxicity induced by oxidative stress, DNA damage, mutagenicity, and the stimulation of inflammatory factors [2]. Our previous study demonstrated that PMs increased oxidative stress and inflammatory responses in A549 cells [3]. However, few studies have focused on the formation of fibrosis, the development of epithelial-mesenchymal transition (EMT) and the related mechanisms caused by PMs exposure. This is the most representative event associated with cell fate and requires attention.

Fibronectin is an important extracellular matrix (ECM) glycoprotein and plays a vital role in the development of fibrosis [4]. The binding of fibronectin and integrin α5β1 (the fibronectin receptor) is an important feature of fibrogenesis [5]. High levels of integrin α5β1 have been found in pulmonary fibrosis of patients with poor prognosis [6]. However, the mechanism associated with PMs-induced pulmonary fibrosis remains unclear. Another important event related to pulmonary fibrosis is PM_2.5_-induced EMT [7]. EMT is the process by which epithelial cells transform into a mesenchymal phenotype and includes the downregulation of epithelial markers, the activation of transcription factors, the upregulation of specific cell surface proteins, the reorganization and expression of cytoskeletal proteins, and the production of ECM-degrading enzymes [8, 9]. Therefore, the molecular mechanisms that regulate the expression of fibronectin and EMT-related proteins may be crucial for the pathogenesis of fibrosis. However, this mechanism has not been studied in detail.

Recent studies have highlighted the important role of transcription factors such as p65 NF-κB in the pathogenesis of EMT and pulmonary fibrosis [10]. Rat type II primary alveolar epithelial cells treated with a p65 inhibitor exhibited reduced levels of placental growth factor-induced EMT [11]. The upregulation of p65 expression may be related to chronic inflammation and EMT and further drive the continuous development of pulmonary fibrosis. In addition, the E26 transformation-specific sequence (ETS) family of transcription factors is increased in extracellular matrix remodeling, which is an important mechanism associated with the pathogenesis of idiopathic pulmonary fibrosis [12]. The loss of the ETS domain-containing protein Elk1 leads to increase integrin α5β6 expression and exacerbate pulmonary fibrosis in an in vivo fibrosis model [13]. The roles of ETS-1 and p-p65 in the pathogenesis of EMT and pulmonary fibrosis have not been determined. In this study, we aimed to investigate EMT and pulmonary fibrosis induced by PMs exposure in vivo and in vitro. To our knowledge, we showed for the first time that PMs exposure induced EMT and fibrosis in a mouse model. We also showed that the expression of ETS-1 and fibronectin is closely related in organic solvent soluble PMs (O-PMs)-treated A549 cells, the lung tissues of PMs-treated mice, and the lung tissues of patients with pulmonary fibrosis.

## Results

### O-PMs induced cell migration and EMT development

**Fig. 1:**
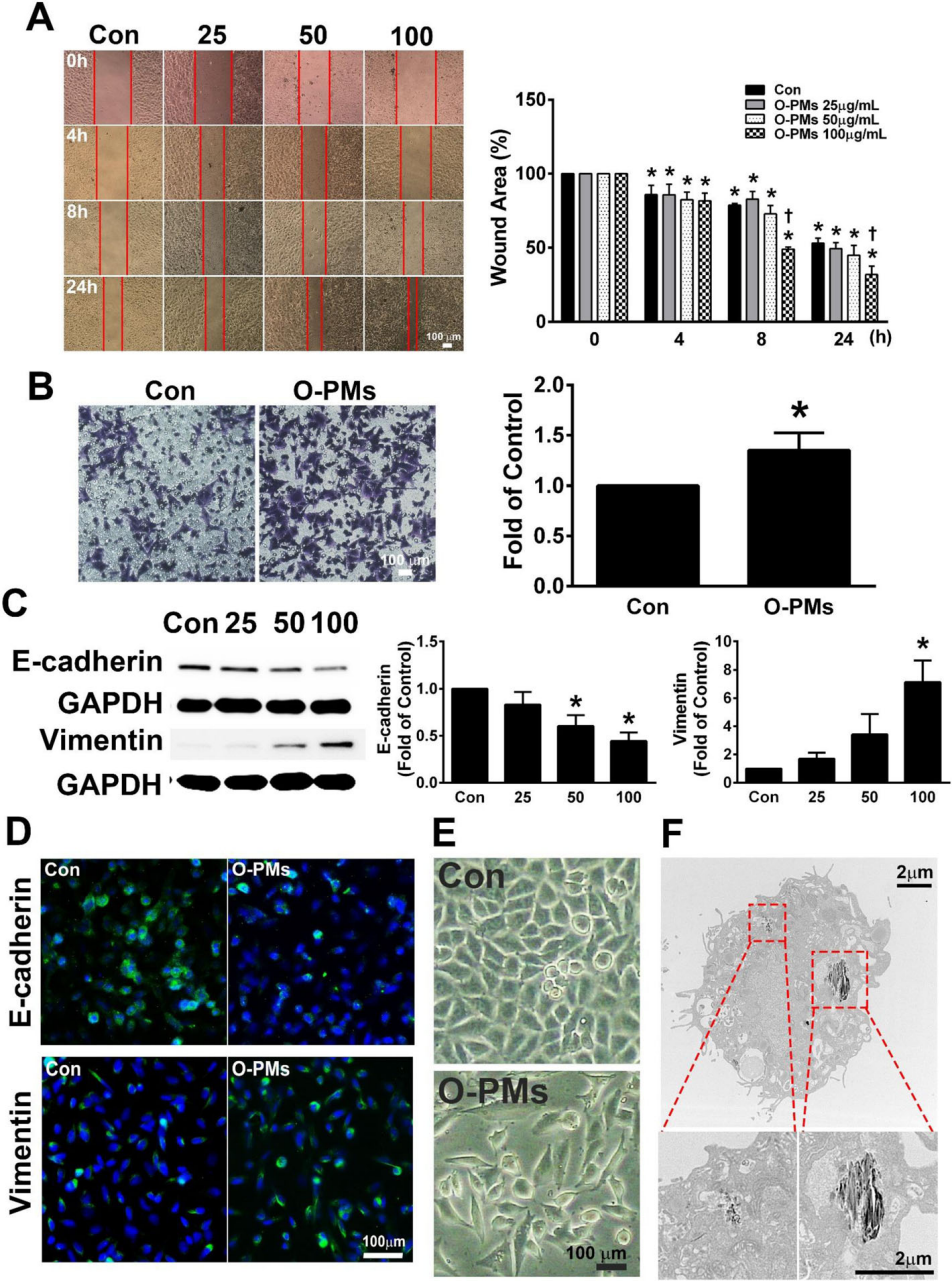
O-PMs induced cell migration and EMT development. **a** The migratory ability of A549 cells treated with 0, 25, 50, and 100 μg/mL O-PMs were measured by wound healing assays. Representative images of each group at different time points after wound formation are shown. The red lines represent the wound boundaries. Bar = 100 μm. The percentage of the wound area is expressed as the mean ± SEM, *n* = 3. **b** A Boyden chamber-based migration assay was used to measure the effect of 100 μg/mL O-PMs on A549 cell migration after 24 h. Representative images of migrating cells are shown. Bar = 100 μm. The quantification of cell migration is expressed as the mean ± SEM. **c** A549 cells were exposed to 0, 25, 50, 100 μg/mL O-PMs for 24 h. The expression levels of E-cadherin and vimentin in the cell lysates were measured by Western blot. GAPDH was used as an internal control. **d** The distribution of E-cadherin and vimentin expression in A549 cells with or without 100 μg/mL O-PMs for 24 h was determined by immunocytochemical staining. Bar = 100 μm. **e** Cells treated with 100 μg/mL O-PMs for 24 h displayed an elongated spindle-like morphology. Bar = 100 μm. **f** PMs particles were present in the cytoplasm, as determined by TEM. Bar = 2 μm. *p < 0.05 compared to control (Con) cells. †*p* < 0.05 compared to the Con group at the same treatment time

To determine whether O-PMs exposure plays an important role in promoting EMT, we examined the concentration- and time- dependence of O-PMs-induced A549 cell migration using a wound healing assay. A549 cells were untreated or exposed to different concentrations of O-PMs for 4, 8, and 24 h, and the wounded areas gradually and significantly decreased in a dose-dependent manner. Importantly, the migration of cells into the wounded area was markedly increased in the presence of 100 μg/mL O-PMs compared to the migration of cells in medium alone at 8 h and 24 h after wounding (Fig. 1a). In addition, the migratory rate was increased in O-PMs-treated cells compared with control cells by the Boyden chamber migration assay (Fig. 1b). To examine whether O-PMs could induce EMT, we measured the effect of O-PMs on EMT markers. A549 cells were incubated with different concentrations of O-PMs for 24 h, and the expression of E-cadherin and vimentin in the cell lysates was determined by Western blot (Fig. 1c). O-PMs treatment decreased E-cadherin expression in a dose-dependent manner compared with that of the control cells (a reduction ratio of 0.6 ± 0.1 at 50 μg/mL and 0.4 ± 0.1 at 100 μg/mL O-PMs). In contrast, O-PMs increased the expression of vimentin in the cell lysates in a dose-dependent manner compared with that of the control group (1.2 ± 0.4 at 25 μg/mL, 3.4 ± 1.5 at 50 μg/mL, and 7.1 ± 1.5 at 100 μg/mL O-PMs). Consistently, fluorescence microscopy analysis showed that E-cadherin was weakly present in O-PMs-treated A549 cells. In contrast, vimentin expression was robust in O-PMs stimulated A549 cells (Fig. 1d). In addition, cells treated with O-PMs displayed an elongated spindle-like morphology (Fig. 1e). PMs particles were present in the cytoplasm, as observed by TEM (Fig. 1f). These results indicated that O-PMs exposure caused significant changes in EMT marker protein expression, suggesting that O-PMs induced EMT in A549 cells.

### O-PMs increased fibronectin expression in A549 cells

**Fig. 2:**
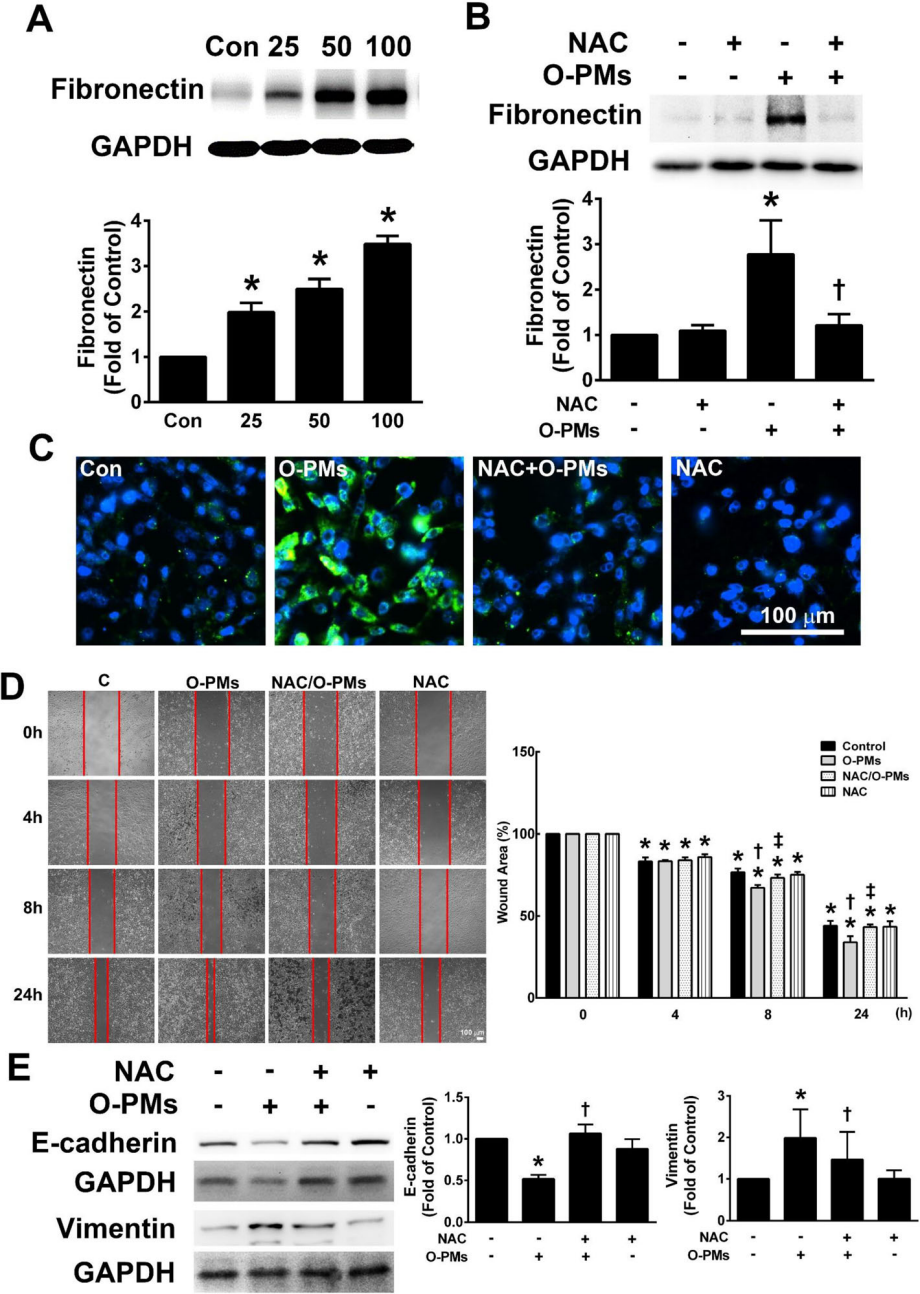
O-PMs increased fibronectin expression in A549 cells. **a** A549 cells were treated with 0, 25, 50, or 100 μg/mL O-PMs for 24 h. Fibronectin expression in the cell lysates was examined by Western blot. **b** A549 cells were treated with or without 5 mM NAC for 1 h, and were then treated with or without 100 μg/ml of O-PMs for 24 h. Fibronectin expression was evaluated by Western blot. **c** The detection and distribution of fibronectin expression in A549 cells with or without 100 μg/mL O-PMs for 24 h was determined by immunocytochemistry. Bar = 100 μm. **d** The effect of NAC on the migratory abilities of O-PMs-treated A549 cells was determined by a wound healing assay. A representative image of each group at different time points after wound formation is shown. The red lines represent the wound boundaries. The percentage of the wound area is expressed as the mean ± SEM, n = 3. Bar = 100 μm. **e** A549 cells were pre-treated with 5 mM NAC and incubated for 1 h. Cells treated with or without NAC were treated with 100 μg/mL O-PMs for 24 h. The expression levels of E-cadherin and vimentin were measured by Western blot. *p < 0.05 compared to control (Con) cells; †*p* < 0.05 compared to O-PMs-treated cells

During EMT, epithelial cell adhesion switches from cell-cell contacts to cell-extracellular matrix interactions, raising the possibility that fibronectin may play a key role in promoting this transition [14]. To examine the effect of O-PMs on fibronectin expression in A549 cells, the cells were treated with 0–100 μg/mL O-PMs for 24 h, and then the expression of fibronectin in the cell lysates was measured by Western blot. As shown in Fig. 2a, O-PMs treatment significantly increased fibronectin expression in a dose-dependent manner (2.0 ± 0.2 at 25 μg/mL, 2.5 ± 0.2 at 50 μg/mL, and 3.5 ± 0.2 at 100 μg/mL O-PMs compared to that of the control). As shown in Fig. 2b, 100 μg/mL O-PMs significantly increased fibronectin expression, while pretreatment with 5 mM N-acetyl cysteine (NAC), an antioxidant, for 1 h attenuated the O-PMs-induced fibronectin expression. These results were consistent with immunofluorescent staining images of fibronectin expression (Fig. 2c). NAC pretreatment markedly reduced O-PMs-induced migration of cells into wounded area (Fig. 2d). A549 cells treated with NAC showed that the O-PMs-induced vimentin expression was decreased, while E-cadherin expression was increased (Fig. 2e). These findings suggest that O-PMs-induced EMT is related to oxidative stress.

### O-PMs increased fibronectin expression via the NF-κB p65 pathway

**Fig. 3:**
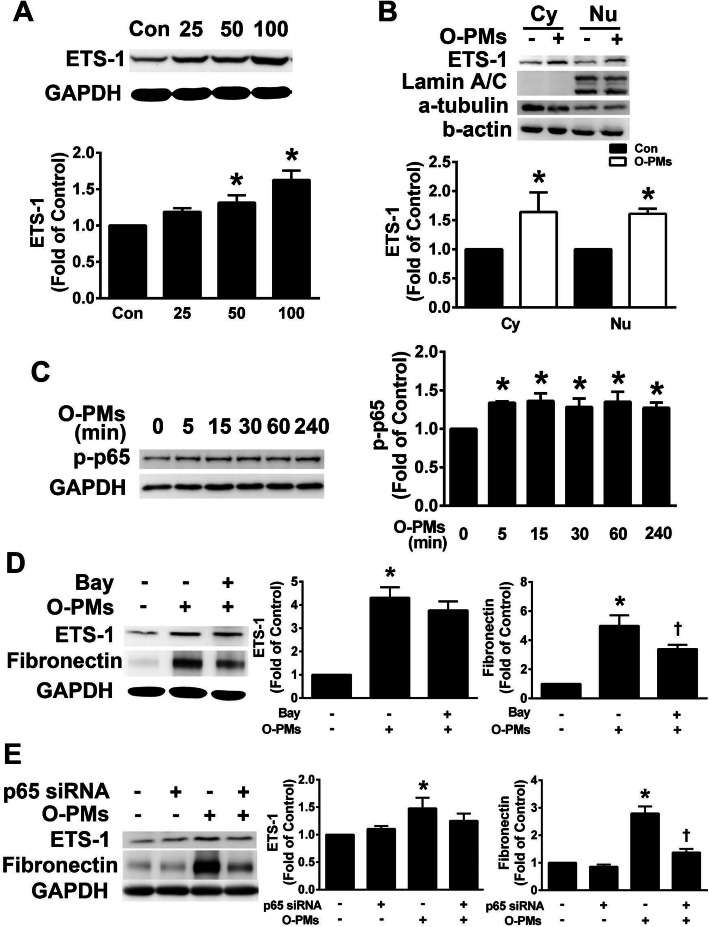
O-PMs increased ETS-1 expression and p65 phosphorylation in A549 cells. **a** A549 cells were exposed to 0, 25, 50, and 100 μg/mL O-PMs for 24 h. The level of ETS-1 in the cell lysates was measured by Western blot. **b** The effect of O-PMs treatment on ETS-1 translocation in A549 cells. A549 cells were treated with or without 100 μg/mL O-PMs for 24 h. The expression of ETS-1 in the cytoplasm (Cy) and nucleus (Nu) was determined by Western blot. **c** A549 cells were treated with 100 μg/mL O-PMs for the specified time. The phosphorylation of NF-κB p65 was measured by Western blot. **d** Cells were pretreated with or without 10 μM Bay11–7082 and then treated with 100 μg/mL O-PMs for 24 h. The expression levels of ETS-1 and fibronectin were determined by Western blot. **e** Twenty-four hours after being transfected with 10 μM p65 siRNA, A549 cells were treated with 100 μg/mL O-PMs for 24 h. The expression levels of ETS-1 and fibronectin were measured by Western blot. *p < 0.05 compared to control (Con) cells; †*p* < 0.05 relative to O-PMs- treated cells

ETS-1 is a transcription factor that is required for EMT [15]. We examined whether the O-PMs-induced fibronectin expression in A549 cells was mediated by the upregulation of ETS-1 and NF-κB p65. As shown in Fig. 3a, O-PMs treatment significantly increased ETS-1 expression in a dose-dependent manner. Next, we explored the effects of O-PMs on the translocation of ETS-1 from the cytoplasm to the nucleus. O-PMs significantly increased the levels of ETS-1 expression in the cytoplasm and the nucleus when compared to that of control cells (Fig. 3b). A previous study reported that NF-κB p65 played an important role in EMT development [10]. O-PMs treatment significantly increased the phosphorylation of NF-κB p65 in A549 cells (Fig. 3c). To further study the role of NF-κB p65 in the expression of ETS-1 and fibronectin in O-PMs-treated cells, we used Bay11–7082 (an NF-κB p65 inhibitor) and p65-specific siRNA transfection to knock down the expression of p65 in A549 cells. Pretreatment with Bay11–7082 reduced the O-PMs-induced increase in fibronectin expression but did not affect the increase in ETS-1 expression (Fig. 3d). A549 cells treated with p65 siRNA exhibited reduced O-PMs-induced fibronectin but not ETS-1 levels, as indicated by Western blot (Fig. 3e). These data suggested that O-PMs increased fibronectin expression via the NF-κB p65 pathway.

### O-PMs affected the expression of fibronectin, E-cadherin and vimentin through the ETS-1 pathway

**Fig. 4:**
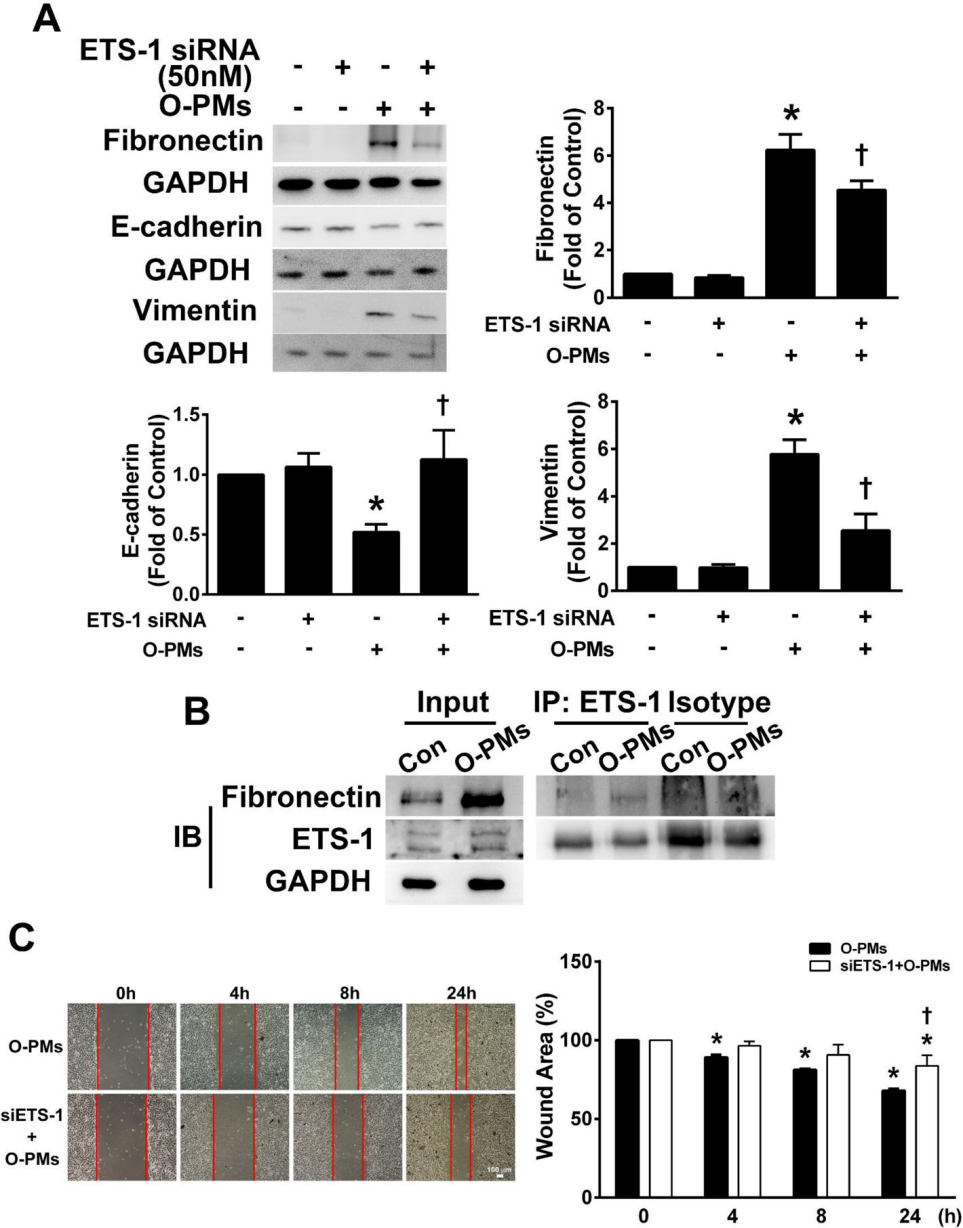
O-PMs affected the expression of fibronectin, E-cadherin and vimentin via the ETS-1 pathway. **a** A549 cells were transfected with 50 nM ETS-1 siRNA and incubated for 24 h. Cells transfected with or without ETS-1 siRNA were treated with 100 μg/mL O-PMs for 24 h. The expression levels of fibronectin, E-cadherin and vimentin were measured by Western blot. **b** Co-immunoprecipitation of ETS-1 and fibronectin in A549 cells. Cell lysates were precipitated with anti-ETS-1 antibody-conjugated agarose beads, and then blotted with anti-ETS-1 and anti-fibronectin antibodies. Fibronectin precipitation was detected in the precipitate of the ETS-1 immune complex. **c** The effect of ETS-1 siRNA on the migratory abilities of O-PMs-treated A549 cells was determined by a wound healing assay. A representative image of each group at different time points after wound formation is shown. The red lines represent the wound boundaries. The percentage of the wound area is expressed as the mean ± SEM, n = 3. Bar = 100 μm. *p < 0.05 compared to control (Con) cells; †*p* < 0.05 relative to O-PMs-treated cells

To further examine the involvement of ETS-1 in the O-PMs-induced fibronectin, E-cadherin and vimentin expression, we used ETS-1 siRNA transfection to knockdown ETS-1 expression in A549 cells. In A549 cells treated with ETS-1 siRNA, the O-PMs-induced increased in fibronectin and vimentin expression were decreased, while E-cadherin expression was increased (Fig. 4a). We next evaluated the interaction of fibronectin and ETS-1 in O-PMs-treated A549 cells. The coimmunoprecipitation results showed that ETS-1 immunoprecipitated with fibronectin, confirming that ETS-1 interacted with fibronectin (Fig. 4b). Pretreatment with ETS-1 siRNA markedly decreased the O-PMs-induced migration of cells into the wounded area (Fig. 4c). These findings suggest that O-PMs-induced EMT is closely related to ETS-1 expression.

### O-PMs-induced EMT is related to the fibronectin receptor

**Fig. 5:**
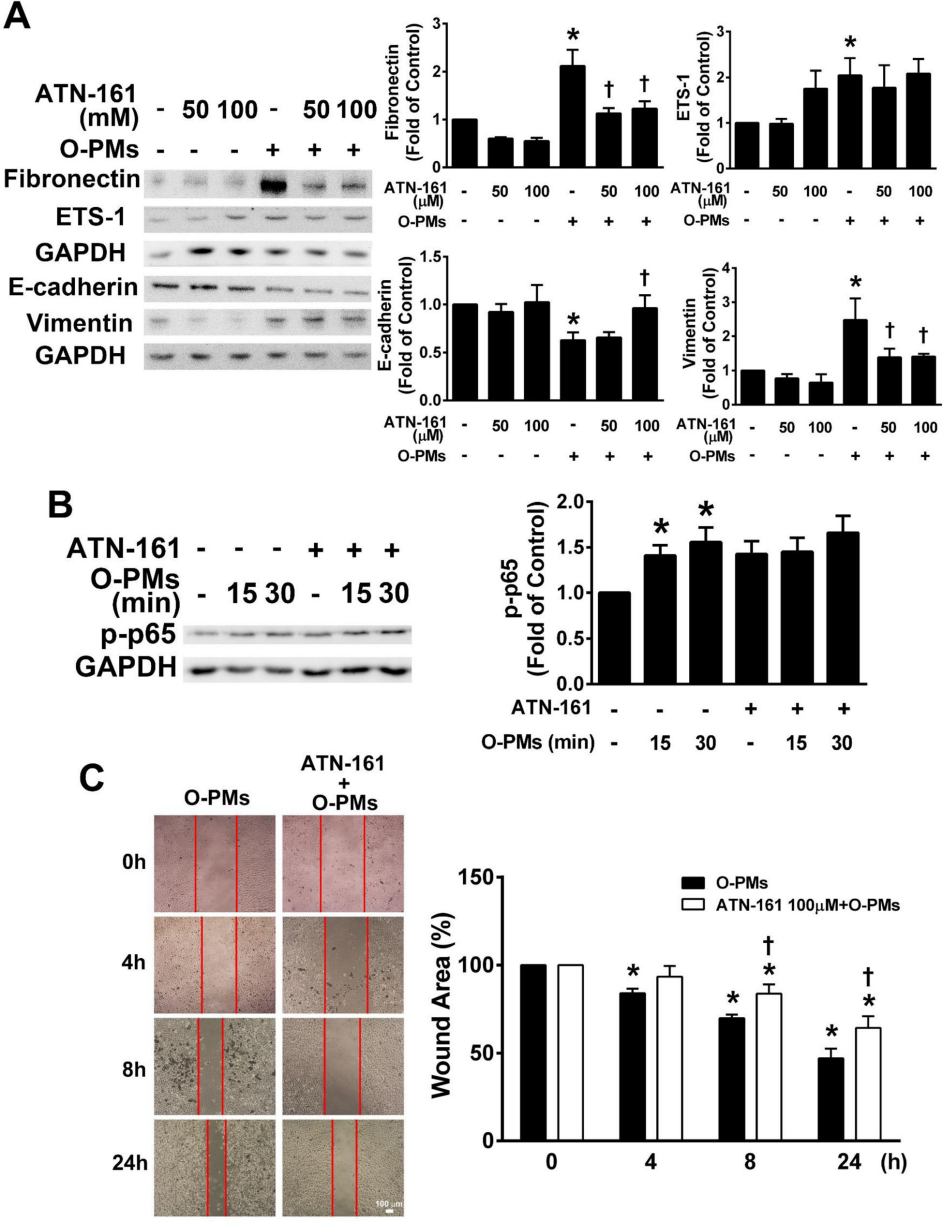
O-PMs-induced EMT is closely related to the fibronectin receptor. **a** A549 cells were pretreated with 50 μM or 100 μM ATN-161 for 30 min, and then treated with 100 μg/mL O-PMs for 24 h. The expression levels of fibronectin, ETS-1, E-cadherin, and vimentin were examined by Western blot. **b** The effect of ATN-161 on the phosphorylation of NF-κB p65 was determined by Western blot. A549 cells were pretreated with 100 μM ATN-161 for 30 min and then treated with 100 μg/mL O-PMs for 15 and 30 min. **c** The effect of ATN-161 on the migratory abilities of O-PMs-treated A549 cells was determined by a wound healing assay. Representative images of each group at the indicated time points after wound formation are shown. The red lines represent the wound boundaries. The percentage of the wound area is expressed as the mean ± SEM, n = 3. Bar = 100 μm. *p < 0.05 vs. control (Con) cells; †*p* < 0.05 vs. O-PMs-treated cells

Fibronectin is recognized by cell surface receptors in integrin family. Integrin α5β1 is particularly efficient in mediating fibronectin matrix assembly [16]. We used ATN-161, a small peptide inhibitor of integrin α5β1, to study the role of the fibronectin receptor in O-PMs-induced EMT. O-PMs significantly increased the expression of fibronectin, ETS-1, and vimentin and decreased E-cadherin expression in A549 cells. In contrast, in cells pretreated with ATN-161, the levels of fibronectin, ETS-1 and vimentin expression were decreased, while E-cadherin expression was increased (Fig. 5a). Interestingly, O-PMs significantly increased the phosphorylation of p65, while ATN-161 had no effect (Fig. 5b). In cells treated with ATN-161 and O-PMs, the migration of the cells into the wounded area was markedly decreased when compared to that of cells treated with O-PMs alone (Fig. 5c). These findings suggest that O-PMs induced EMT is closely related to the fibronectin receptor, and blocking the fibronectin receptor can downregulate ETS-1 expression and reverse O-PMs-induced EMT.

### The effects of PMs on collagen deposition and EMT-related proteins in lung tissues

**Fig. 6:**
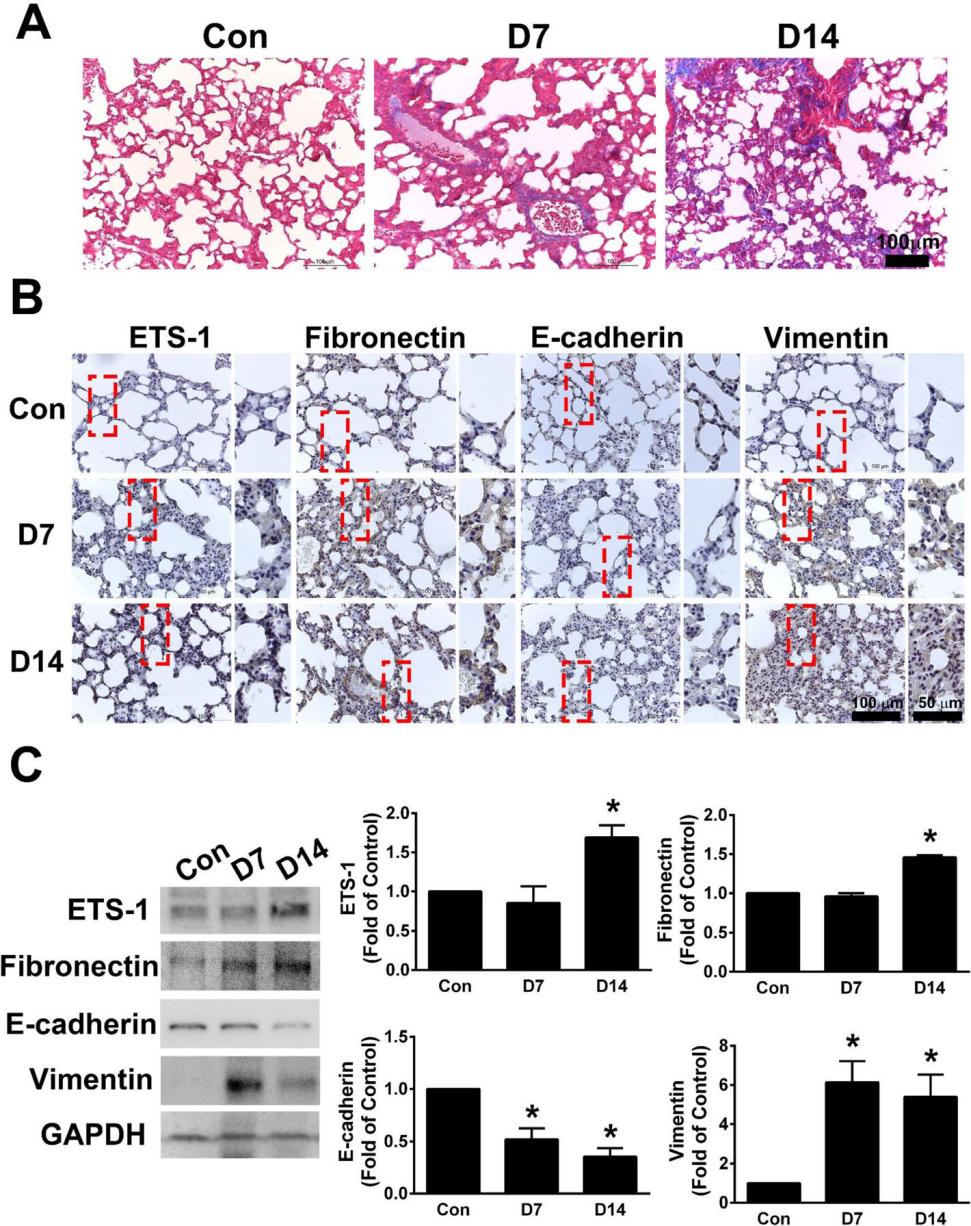
PMs induced collagen deposition and EMT-related protein expression in lung tissues in vivo. WT mice were treated with 200–350 μg/mouse PMs by intratracheal instillation. After 7 or 14 days, the mice were euthanized and lung tissue was collected. **a** Collagen deposition in lung tissue was determined by Masson’s trichrome staining. Blue: collagen. Bar = 100 μm. **b** The expression of ETS-1, fibronectin, E-cadherin and vimentin in mouse lung tissue was measured by immunohistochemical staining. The dotted rectangle in the left image is enlarged and displayed in the right image. **c** Western blot was used to measure the expression levels of ETS-1, fibronectin, E-cadherin and vimentin in mouse lung tissue. Bar = 100 μm. *p < 0.05 vs control (Con) group

**Fig. 7:**
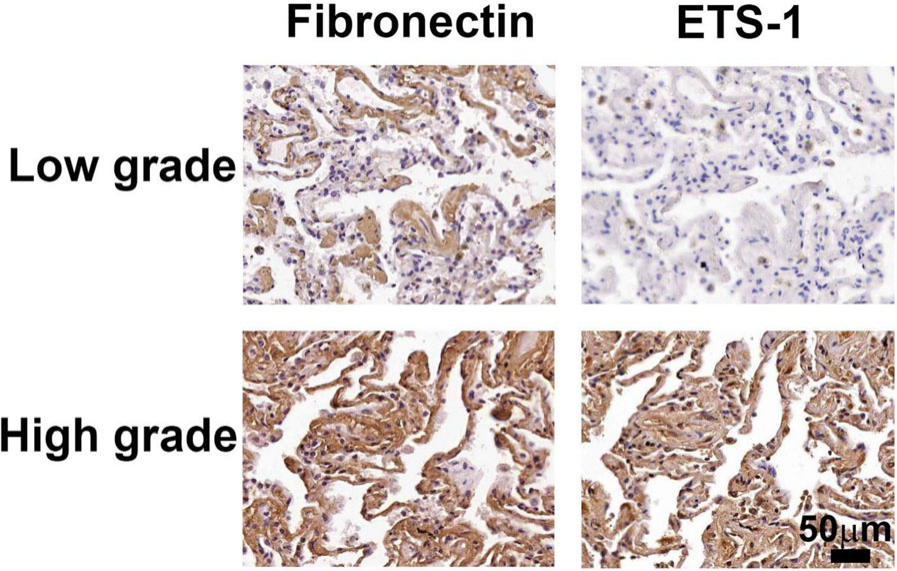
Representative images of immunohistochemical staining of ETS-1 and fibronectin in a human pulmonary interstitial fibrosis tissue microarray. Bar = 50 μm

To detect the effects of PMs on EMT in vivo, WT mice were untreated or injected intratracheally with PMs in PBS (200–350 μg/mouse) for 7 or 14 days. The lung tissues were examined by Masson’s-trichrome staining, Western blot and immunohistochemical staining. As shown in Fig. 6a, PMs significantly induced collagen deposition in the perivascular region in lung tissue at Day 7 and the perialveolar region in lung tissue at Day 14. PMs significantly induced the expression of ETS-1, fibronectin, and vimentin in lung tissues and decreased the expression of E-cadherin at Day 7 and Day 14, as detected by Western blot and immunohistochemical staining at Day 7 and Day 14 (Fig. 6b and c). These findings suggested that fibrosis and EMT were present in the lung tissues of mice after exposure of PMs on Day 7 and Day 14. Furthermore, we examined the relationship between ETS-1 and fibronectin expression in human pulmonary interstitial fibrosis by using a human tissue microarray. The different levels of ETS-1 and fibronectin expression, as detected by immunohistochemical staining, are shown in Fig. 7. Table 1 shows a significant correlation between ETS-1 and fibronectin levels in pulmonary interstitial fibrosis.

**Table 1:** Correlation between ETS-1 expression and fibronectin expression in a human lung interstitial fibrosis tissue microarray. In tissue sections, the percentage of both ETS-1 and fibronectin expression was 53.3%; the expression of ETS-1 or fibronectin alone was 46.6% or 15.4%, respectively. The percentage of neither protein was 84.6%

Total *n* = 28		ETS-1 expression		*p-*value
		Positive	Negative	
Fibronectin expression	Positive	8 (53.3%)	2 (15.4%)	0.0366
	Negative	7 (46.6%)	11 (84.6%)	

## Discussion

This study clearly demonstrated that O-PMs can induce EMT development and fibronectin expression through the nuclear transcription factors ETS-1 and NF-κB p65 in A549 cells. The expression of EMT markers, fibronectin, ETS-1 and pulmonary fibrosis was observed in PMs-treated mice. A significant correlation between fibronectin and ETS-1 level was also observed in human lung fibrotic tissue. The most important and novel finding is that the ETS-1 pathway may be important in the pathogenesis of EMT and pulmonary fibrosis.

EMT is characterized by the lose of cell-cell adherens junctions and apical-basal polarity and the acquisition of mesenchymal features with a spindle-like cell shape and migratory abilities [17]. EMT may be active in the pathogenesis of COPD, airway fibrosis and lung cancer [18]. Cigarette smoke extract induced the cytotoxicity of airway epithelial cells and changed EMT markers such as E-cadherin, N-cadherin, and vimentin [19]. Our present study showed that O-PMs-induced alveolar epithelial cells exhibited increased vimentin expression and downregulated E-cadherin expression, as well as alterations from epithelial to spindle-like mesenchymal morphology. The PMs used in this study was from SRM 1649b, and the certificate of analysis has been previously reported [20]. The chemical composition of SRM 1649b included water-soluble and organic extractable fractions, which have biological and toxicological effects [21]. The extractable organic fraction contained polycyclic aromatic hydrocarbons (PAHs), steranes and hop alkanes, which can produce increased levels of ROS and induce cytotoxic and inflammatory effects [22, 23]. In addition, organic extractable fractions can trigger a cascade of intracellular signaling (e.g., IL-6) and PAH-related aryl hydrocarbon receptor (AhR)-dependent signaling (e.g., CYP1A1) [3, 22]. Our previous study demonstrated that O-PMs significantly increased ICAM-1 expression in alveolar epithelial cells through an IL-6/AKT/STAT3/NF-κB-dependent pathway [3]. Furthermore, the present study demonstrated that O-PMs-treated cells exhibited increased migration abilities in a dose-dependent manner by a scratch wound healing assay and fibronectin expression. Our results suggest that EMT is involved in the transformation of A549 cells and lung tissues by PMs.

The expression of EMT-related cytokines is regulated by transcription factors [24]. Phosphorylation of the transcription factor NF-κB is associated with inflammation and respiratory diseases caused by cigarette smoke [25–27]. In addition, ROS-activated NF-κB is also associated with the phenotypic transformation of epithelial cells [28, 29]. The organic extract of PM_2.5_ enhanced the binding of NF-κB to the promoter of long noncoding RNA metastasis-associated lung adenocarcinoma transcript 1 (lncRNA MALAT1) and caused a mesenchymal phenotypic change in lung bronchial epithelial cells [30]. Consistent with previous reports, we found that O-PMs-induced NF-κB activation in A549 cells. The NF-κB inhibitor BMS-345541 could abrogate the increase in fibronectin deposition in lung fibroblasts isolated from COPD patients after stimulation with cigarette smoke extracts and TGF-β [31]. Our results further demonstrated that the use of p65-siRNA and the NF-κB inhibitor Bay 11–7082 eliminated the expression of fibronectin. Based on these results, we concluded that O-PMs increased the expression of fibronectin through the NF-κB pathway.

The pathogenesis of fibrosis is closely related to the expression of ETS-1 [32]. The expression of ETS-1 mRNA is related to the EMT phenotype, which is characterized by vimentin expression and E-cadherin deficiency in breast cancer cell lines [33]. However, the effect of ETS-1 on the expression of vimentin, E-cadherin and fibronectin, which are involved in the EMT associated phenotype, has not been studied in alveolar epithelial cells. TGF-β1 induced ETS-1 expression through p38 MAPK signal in renal epithelial cells [34]. Therefore, whether PM2.5 affects ETS-1 expression and its signal transduction needs to be clarified. In the present study, we demonstrated that ETS-1 was significantly expressed in a dose-dependent manner in A549 cells treated with O-PMs. Our data also showed that ETS-1 silencing reduced vimentin expression and restored E-cadherin expression in O-PMs-treated cells. In addition, our results indicated that inhibition of ETS-1 could reduce the expression of fibronectin, which was similar to previous findings that angiotensin II induced fibronectin expression and renal fibrosis through ETS-1 [32]. We further demonstrated that at 7 or 14 days after intratracheal injection of PMs, the expression of fibronectin and ETS-1 in mouse lung fibrous tissue increased significantly. In this study, a significant correlation between fibronectin and ETS-1 expression was further demonstrated in the tissue array analysis of patients with pulmonary interstitial fibrosis. Therefore, by demonstrating the close relationship between ETS-1 and EMT-related molecules, we provide strong evidence that ETS-1 expression plays a vital role in the development of EMT in O-PMs-treated alveolar epithelial cells.

Matrix-specific integrin signals may contribute to multiple processes during EMT development and pulmonary fibrosis [35]. The previous report has shown that fibronectin can increase endothelial activation in response to a variety of atherosclerotic stimuli, and limiting fibronectin deposition can alleviate early inflammation in atherosclerotic plaques [36]. The fibronectin receptor α5β1 integrin mediates oxidized low-density lipoprotein-induced inflammation and atherosclerosis [37]. Integrin α5β1 has recently been considered to be the main mediator of tumor angiogenesis [38]. Treatment with the α5β1 integrin inhibitor ATN-161 can prevent the growth of breast cancer and reduce the density of microvessels in the body [39]. Our current study showed that ATN-161 significantly reduced the expression of fibronectin and vimentin, and increased the expression of E-cadherin. ATN-161 treatment limited the formation of fibrosis and the development of EMT. However, treatment of cells with ATN-161 did not affect O-PMs-induced ETS-1 and p-p65 expression levels. Our data suggest that the α5β1 integrin inhibitor, which is currently in clinical trials for cancer [39], can be used to treat EMT and pulmonary fibrosis.

## Conclusion

**Fig. 8:**
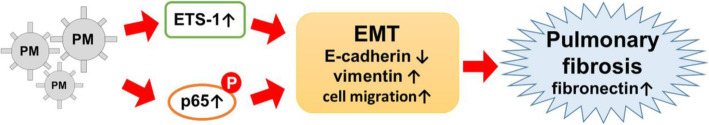
Schematic of O-PMs-induced the formation EMT and fibrosis and the related mechanisms

The current results show that O-PMs can induce EMT and fibronectin expression by activating the transcription factors ETS-1 and NF-κB in A549 cells (Fig. 8). PMs can induce expression of EMT, fibronectin and ETS-1 in mouse lung tissue. A significant correlation between fibronectin and ETS-1 can also be seen in human lung fibrotic tissue. All these findings suggest that the ETS-1 pathway may be a novel alternative pathway for EMT formation and pulmonary fibrosis. This new ETS-1 pathway that induces EMT and fibronectin expression is highly correlated with α5β1-integrin activation.

## Material and methods

### Preparation of PMs dissolved in an organic solvent (O-PMs)

Standard reference material 1649b (SRM 1649b; PM) was purchased from the National Institute of Standards and Technology (NIST; MD, USA) and was prepared from atmospheric particulate material collected in the Washington, DC area in 1976 and 1977 using a specially designed baghouse. The PM was collected over a period longer than 12 months and represents a time-integrated sample. A total of 100 mg of PMs was dissolved in 1 mL of the organic solvent dimethyl sulfoxide (Sigma, MO, USA) and used after vortexing. O-PMs were stored at 4 °C for subsequent experiments.

### Cell culture

A549 cells (neoplastic, transformed of human lung type II epithelial cells) were purchased from the American Type Culture Collection (ATCC, VA, USA) and cultured in Dulbecco’s Modified Eagle Medium (DMEM, Biological Industries, CT, USA) containing 10% fetal bovine serum (FBS, Biological Industries) and 1% Penicillin/Streptomycin/Amphotericin B (Biological Industries). Cells were grown in a humidified incubator at 37 °C (5% CO_2_/95% air atmosphere.

### Wound healing assay

To determine whether O-PMs affect cell migration, the cell monolayer was scraped with a sterile 200 μL pipette tip and then treated with or without 25, 50, or 100 μg/mL of O-PMs. The cells were observed under the microscope and photographed at the designated times (0, 4, 8, 24 h). The percentage of the wound closure area/original wound area was calculated using ImageJ software.

### Transwell migration assay

Boyden chambers (8-μm pore size; Millipore, MA, USA) were used to examine the effects of O-PMs on cell motility. A549 cells were placed in the upper chamber and treated with or without 100 μg/mL O-PMs for 24 h. The migrated cells that were attached to the lower surface of the membrane were stained with 2% crystal violet in 2% ethanol. The cells were photographed and counted by ImageJ.

### Cell lysate preparation and Western blot analysis

Cells were treated with or without 25, 50, or 100 μg/mL of O-PMs and harvested with RIPA buffer (H.M. Biological, Taoyuan, Taiwan) supplemented with protease and phosphatase inhibitors (Thermo Fisher Scientific, MA, USA). In addition, cytoplasmic and nuclear proteins were extracted using a Nuclear Extraction Kit (Cayman Chemical, MI, USA). Thirty micrograms of protein were subjected to sodium dodecyl sulfate polyacrylamide gel electrophoresis (SDS-PAGE). The membranes were incubated overnight at 4 °C with primary antibodies against fibronectin, ETS-1 (1: 2000 dilution, Abcam, Cambridge, UK), E-cadherin, phosphorylated-NF-κB p65 (1:2000 dilution, Cell Signaling Technology, MA, USA), Lamin A + C, α-tubulin, β-actin (1:2000 dilution, GeneTex, CA, USA), or vimentin (1:2000 dilution, Santa Cruz Biotechnology, TX, USA). The anti-GAPDH antibody (1:10000 dilution, Tools, New Taipei City, Taiwan) was used as the loading control. Images were visualized by UVP BioSpectrum 815 imaging system (UVP, CA, USA), and the intensity of each band was quantified using ImageJ software.

### Immunocytochemistry

To examine the effect of O-PMs on the in situ expression of EMT markers and fibronectin, confluent A549 cells on sterilized-coverslip in 12-well plate were incubated with 1 mL DMEM medium containing 10% FBS with or without adding 100 μg/mL of O-PMs for 24 h by immunocytochemistry. The cells were fixed in 4% paraformaldehyde, permeabilized with 0.01% Triton X-100, blocked with 1% bovine serum albumin (BSA) in PBS, and then incubated with the indicated primary antibodies at 4 °C overnight. After being washed with PBS, the cells were incubated with AlexaFluor 488 conjugated secondary antibodies (Abcam), and then observed and photographed with a fluorescence microscope. DAPI was used for nuclear counterstaining.

### Transmission electron microscopy (TEM)

A549 cells were treated with 100 μg/mL O-PMs for 24 h, collected by centrifugation, washed with PBS, fixed with 2% glutaraldehyde and 2% paraformaldehyde in PBS for 1 h, and postfixed with 1% osmic acid for 30 min. The samples were then dehydrated in graded ethanol, washed with propylene Oxide and embedded in epoxy resin. Ultrathin sections were cut in a Reichert ultramicrotome, stained with lead citrate and uranyl acetate and examined with a HITACHI H-7100 at 100 kV.

### siRNA transduction

To examine whether the expression of ETS-1 and p65 is involved in the EMT process, specific siRNA obtained from GenePharma (Shanghai, China) or Santa Cruz Biotechnology siRNA were used to target and silence ETS-1 or p65, respectively. A549 cells were treated with 50 nM ETS-1 siRNA or 10 nM p65 siRNA in TurboFect™ transfeection reagent (Thermo Fisher Scientific). Twenty-four hours after siRNA transfection, the cells were stimulated for another 24 h with or without 100 μg/mL O-PMs. The downregulation of EMT-related proteins in cell lysates was examined by Western blot.

### Co-immunoprecipitation assay

To further examine the relationship between ETS-1 and fibronectin in A549 cells after O-PMs exposure, A549 cells treated with or without 100 μg/mL O-PMs for 24 h were lysed in 0.5 mL of lysis buffer (50 mM Tris-HCL, pH 7.4, 150 mM NaCl, 0.1% Triton X-100, and 0.1% SDS), then incubated with a 50% slurry of GammaBind Plus-Sepharose (BD Biosciences, CA, USA) conjugated with the indicated antibodies at 4 °C overnight. The precipitated proteins were subjected to Western blot.

### Animal model of intratracheal instillation of PMs

To test the effect of PMs on EMT in vivo, 8- to 12-week-old male C57BL/6 wild-type (WT) mice weighing 25–35 g were purchased from National Taiwan University, Taiwan. The mice were divided into three groups according to treatment and time after PMs exposure: (1) control group without PMs treatment, (2) mice assessed 7 days after PMs injection, and (3) mice assessed 14 days after PMs treatment. The mice were anesthetized with inhaled 2% isoflurane, the trachea was exposed, and then an insulin syringe was used to puncture the anterior wall of the trachea at a 45° angle to avoid damage to the posterior wall.

A 100 μL suspension containing 200–350 μg of PMs in sterile PBS was slowly instilled intratracheally. The dose range of PMs was based on the body weights (10 mg/Kg) of the mice. The mice were sacrificed on the 7th (D7) or 14th day (D14) after intratracheal instillation. A portion of the lung tissue was immersed in 4% buffered paraformaldehyde for fixation and embedded in paraffin for immunohistochemistry. The remaining portion was immediately frozen in liquid nitrogen for Western blot analysis.

All procedures involving experimental animals were conducted in accordance with the guidelines for animals of National Taiwan University (IACUC No. 20160235) and complied with the Guide for the Care and Use of Laboratory Animals (NIH publication no. 86–23, revised 1985).

### Masson’s trichrome staining

To determine the collagen levels in the lung tissues of mice after PMs injection, we performed a modified Masson staining according to the manufacturer’s instructions (ScyTek Laboratories, UT, USA). The control (Con), day 7 (D7), and day 14 (D14) sections were observed using a light microscope.

### Immunohistochemistry

To determine the levels of E-cadherin, vimentin, fibronectin, and ETS-1 in lung tissues after PMs injection, 5-μm-thick sections were incubated with the indicated antibodies (1:200 dilution) at 4 °C overnight. After incubation with biotin-conjugated secondary antibodies, the sections were stained with 3, 3-diaminobenzidine tetrahydrochloride (DAB; Vector laboratories, CA, USA), counterstained with hematoxylin, and then examined with a light microscope.

### Human tissue microarray

The relationship between ETS-1 and fibronectin in human lung fibrosis is still unknown and needs to be clarified. The human lung interstitial fibrosis tissue microarray (LC561) used in this study was purchased from US Biomax Inc. (MD, USA). The levels of ETS-1 or fibronectin expression were quantified by pathologists at GenDiscovery (New Taipei City, Taiwan). A pathologist at GenDiscovery (New Taipei City, Taiwan) quantified the expression level of ETS-1 or fibronectin.

### Statistical analysis

All data are expressed as the mean ± SEM. The difference between the experimental group and the control group was evaluated by Student’s t-test, and a value of *p*<0.05 was considered statistically significant. (*p<0.05 compared with the control group; †*p* < 0.05 compared with the O-PMs). The correlation between ETS-1 and fibronectin expression in the human tissue array was determined by the chi-square test.

## Data Availability

All the data and materials are available. These are all openly available.

## Abbreviation

O-PMs: Organic solvent-soluble PMs
EMT: Epithelial-mesenchymal transition
ETS-1: E26 transformation-specific sequence-1
COPD: Chronic obstructive pulmonary disease
BSA: Bovine serum albumin
DMEM: Dulbecco ‘s Modified Eagle Medium
DMSO: Dimethyl sulfoxide
FBS: Fetal bovine serum
NAC: N-acetyl cysteine
PAHs: Polycyclic aromatic hydrocarbons
SDS: Sodium dodecyl sulfate
ROS: Reactive oxygen species
AhR: Aryl hydrocarbon receptor
